# Joint Learning of Binocularly Driven Saccades and Vergence by Active Efficient Coding

**DOI:** 10.3389/fnbot.2017.00058

**Published:** 2017-11-03

**Authors:** Qingpeng Zhu, Jochen Triesch, Bertram E. Shi

**Affiliations:** ^1^Department of Electronic and Computer Engineering, Hong Kong University of Science and Technology, Hong Kong, Hong Kong; ^2^Frankfurt Institute for Advanced Studies, Frankfurt am Main, Germany

**Keywords:** active efficient coding, saccades, vergence, binocular saliency map, generative adaptive subspace self-organizing map, reinforcement learning

## Abstract

This paper investigates two types of eye movements: vergence and saccades. Vergence eye movements are responsible for bringing the images of the two eyes into correspondence, whereas saccades drive gaze to interesting regions in the scene. Control of both vergence and saccades develops during early infancy. To date, these two types of eye movements have been studied separately. Here, we propose a computational model of an active vision system that integrates these two types of eye movements. We hypothesize that incorporating a saccade strategy driven by bottom-up attention will benefit the development of vergence control. The integrated system is based on the active efficient coding framework, which describes the joint development of sensory-processing and eye movement control to jointly optimize the coding efficiency of the sensory system. In the integrated system, we propose a binocular saliency model to drive saccades based on learned binocular feature extractors, which simultaneously encode both depth and texture information. Saliency in our model also depends on the current fixation point. This extends prior work, which focused on monocular images and saliency measures that are independent of the current fixation. Our results show that the proposed saliency-driven saccades lead to better vergence performance and faster learning in the overall system than random saccades. Faster learning is significant because it indicates that the system actively selects inputs for the most effective learning. This work suggests that saliency-driven saccades provide a scaffold for the development of vergence control during infancy.

## Introduction

Biological vision systems are often active and rely on a number of eye movements to sense the environment. Remarkably, these vision systems have the ability to autonomously self-calibrate, but the underlying mechanisms are still poorly understood. Here, we focus on vergence and saccadic eye movements. Vergence eye movements are slow and disconjugate (the two eyes move in opposite directions). They serve to align the images acquired by the two eyes so that they can be binocularly fused. Saccadic eye movements are rapid and conjugate (the two eyes move in the same direction). They serve to direct gaze so that the fovea, the region with highest visual acuity, falls on objects of interest. The two types of eye movements often cooccur. For example, they are both involved when eye movements are made to direct gaze toward different objects in a 3D scene (Yang et al., 2002). The association between vergence and saccades facilitates the imaging of objects of interest onto the fovea of both eyes (Zee et al., 1992).

Saccades are of great importance for human vision. At any time, the visual system receives a large amount of information from the environment, but has limited capacity for sensing and signal processing. Humans use saccades to direct foveal vision toward places with relevant information (Yarbus, 1967; Renninger et al., 2007). These saccades can be driven by top-down (Gao et al., 2009; Kanan et al., 2009; Yang and Yang, 2012) or bottom-up (Itti et al., 1998; Hou and Zhang, 2007; Zhang et al., 2008; Bruce and Tsotsos, 2009; Han et al., 2011) attention mechanisms. Top-down attention is voluntary and task-driven, whereas bottom-up attention is involuntary and stimulus-driven. We focus here on the bottom-up mechanism, where saccades are assumed to be generated according to a saliency map, which assigns salience to different points on an image by combining a number of low-level features. For example, Itti et al. (1998) proposed to generate a saliency map by combining the outputs of feature maps that are sensitive to different features, such as color, intensity, and orientation. Since the primary visual cortex (area V1) is one of the first stages of visual information processing, many models have been inspired by the processing found there. For example, Li (2002) proposed to generate the saliency map by combining the responses of model V1 neurons tuned to input features such as orientation and color. The attention based on information maximization (AIM) saliency model, proposed by Bruce and Tsotsos (2009), combines feature maps generated by a set of learned basis functions that are similar to the receptive fields of V1 neurons.

Most proposed saliency models, including those described earlier, assume monocular images, ignoring the importance of depth in human vision. Depth cues play an important role in visual attention (Wolfe and Horowitz, 2004) and have a strong relationship with objects, since depth discontinuities suggest object boundaries. Although some saliency models have incorporated depth cues, they have typically processed depth and 2D texture information separately, e.g., by combining saliency maps computed by considering each cue in isolation. For example, Wang et al. (2013) proposed a visual saliency model that combines a saliency map computed from disparity with a saliency map computed from monocular visual features. Liu et al. (2012) proposed a saliency model where disparity information is extracted by taking the difference between the left and right images. They compute the overall saliency as the weighted average of the saliencies computed from disparity, color, and intensity separately.

We describe here an integrated vision system that combines binocular vergence control and binocularly driven saccadic eye movements. This system extends prior work on learning binocular vergence control using the active efficient coding (AEC) framework, first proposed by Zhao et al. (2012). The AEC framework is an extension of Barlow’s (Barlow, 1961) efficient coding hypothesis, which states that the activity of the sensory-processing neurons encodes their input using as few spikes as possible. A primary prediction of the efficient encoding hypothesis is that the properties of the sensory-processing neurons adapt to the statistics of the input stimuli. The AEC framework extends the efficient coding hypothesis to include the effect of behavior. It posits that in addition, the organism’s behavior adapts so that the input can be efficiently encoded. By combining unsupervised and reinforcement learning, AEC simultaneously learns both a distributed representation of the sensory input and a policy for mapping this representation to motor commands. Thus, it jointly learns both perception and action as the organism behaves in the environment. In previous work, AEC has been shown to model the development of many reflexive eye movements and other behaviors, such as vergence control (Zhao et al., 2012; Lonini et al., 2013; Klimmasch et al., 2017), smooth pursuit (Zhang et al., 2014; Teulière et al., 2015), optokinetic nystagmus (Zhang et al., 2016), the combination of vergence and smooth pursuit (Vikram et al., 2014), and imitation learning (Triesch, 2013).

We make several contributions in this work. First, in the original work by Zhao et al. (2012), saccades were generated completely randomly. This paper integrates the vergence control process with a more realistic model of saccade generation. Second, we extend Bruce and Tsotsos’s (Bruce and Tsotsos, 2009) AIM saliency model, which was formulated for monocular images, to binocular images. In particular, instead of using a set of fixed pre-trained monocular basis functions learned on a separate database, our model uses a set of binocular basis functions that are learned as the model agent interacts with the environment. These low-level binocular features integrate depth and texture information much earlier than in the prior work described earlier and are consistent with what is known about the visual cortex. Poggio and Fischer (1977) claimed that most cortical neurons (84%) are sensitive to the depth of a stimulus. Third, we propose a saliency model, where the saliency of a given point depends upon the current fixation point, whereas most prior saliency models have assigned saliency independently of the current fixation point. In this version of the saliency model, image points that are different from the current fixation point in terms of appearance or depth are more salient. Fourth, rather than treating saliency and vergence control as two separate problems, our model exhibits a very close coupling between the two. Not only are the two behaviors learned at the same time but they also share the same set of low-level feature detectors.

## Materials and Methods

### Architecture Overview

**Figure 1:**
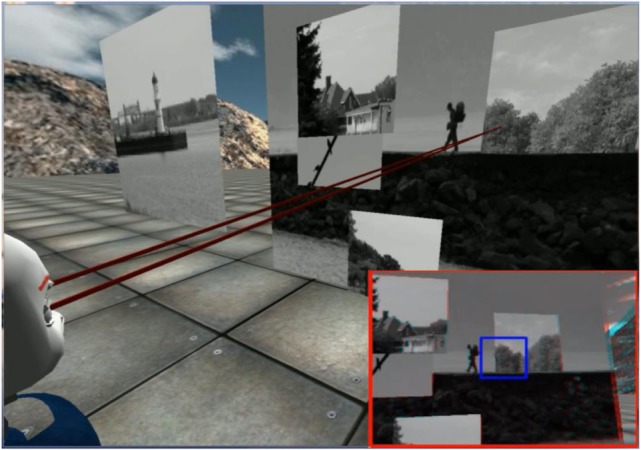
The virtual environment in the iCub simulator. The two red rays indicate the eye gaze vectors. The inset at the lower right hand corner outlined in red shows a red-cyan anaglyph of the stereo images.

Our model assumes the robot is in an environment that has multiple objects located at different depths. We have made a video^1^ to demonstrate how our system works in the iCub simulator (Tikhanoff et al., 2008). One frame of the video is shown in Figure 1. The system drives the robot eyes to saccade to different fixations chosen according to a probability distribution over the points in the scene. This probability distribution is derived from the saliency map of the scene. Fixations last 400 ms. During each fixation, the system controls the vergence eye movement. After each fixation, the robot saccades to another fixation point in the environment.

**Figure 2:**
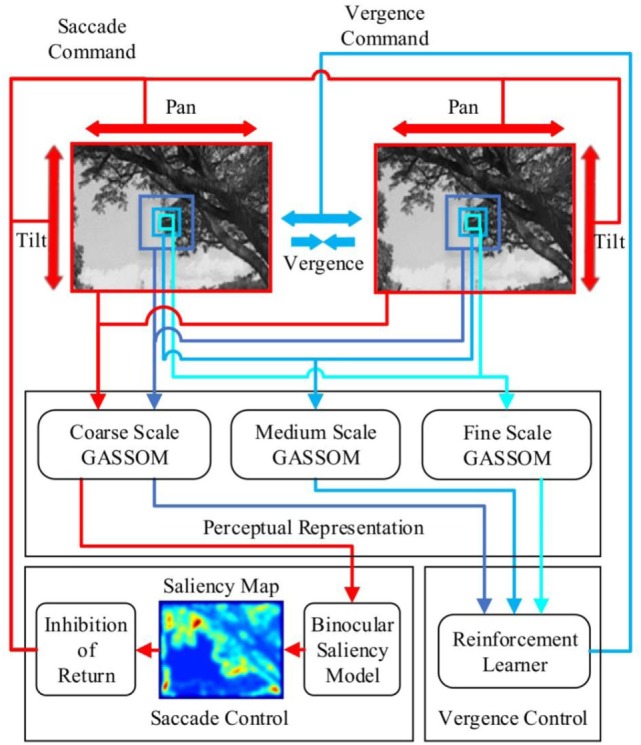
The architecture of the model integrating vergence and saccadic eye movements. Red regions in the saliency map correspond to high values while blue regions correspond to low values. The red arrows identify the steps in generating the saccade command. The blue arrows identify the steps in generating the vergence command.

The architecture of the integrated active vision system is illustrated in Figure 2. It consists of three main parts: the perceptual representation mechanism, the saccade control mechanism, and the vergence control mechanism.

The inputs to the system come from pairs of sub-windows from the left and right camera images. The input to the saccade control mechanism comes from the largest pair of sub-windows, which cover most of the images. This pair is down-sampled to generate a coarse scale representation. The input to the vergence control mechanism comes from three pairs of sub-windows: a pair of small fine scale sub-windows, a pair of medium-sized medium scale sub-windows, and a pair of large coarse scale sub-windows.

The perceptual representation mechanism encodes the binocular image inputs using a distributed representation learned using the generative adaptive subspace self-organizing map (GASSOM) algorithm (Chandrapala and Shi, 2014). The GASSOM model is a statistical generative model for time-varying sensory input that combines both sparsity and slowness. The same perceptual representation is used to generate the input to both the saccade and the vergence control mechanisms.

The vergence control mechanism maps the GASSOM representation of the binocular inputs at all three scales to a set of discrete vergence actions. The vergence action is chosen according to a probability distribution computed using a neural network with a softmax output. The image input in the next iteration changes because of the vergence action.

The saccade control mechansim maps the GASSOM representation of the entire left and right eye images at the coarse scale to a fixation point by sampling the fixation point from a 2D probability distribution generated by a binocular saliency map. The saliency control policy also includes inhibition of return (IOR) to prevent the system from returning to a previously generated fixation too quickly. Saccades move the eyes to the selected fixation point while keeping the vergence angle the same.

Below, we describe in more detail the experimental setup and the three parts of the integrated active vision system. To avoid clutter in the notation, we do not indicate time explicitly. However, it should be understood that most quantities evolve over time either due to the agent’s behavior in the environment, e.g., the inputs and actions, or due to learning, e.g., the network weights. Both behavior and learning progress simultaneously.

### Experimental Setups

We trained and tested our system in two different simulation environments: the Tsukuba environment and the iCub environment. Most experiments are implemented in the Tsukuba environment. The results of experiments using the iCub environment are reported in Figures 5 and 10.

The Tsukuba environment is based on the Tsukuba dataset (Martull et al., 2012), which contains 1,800 photorealistic stereo image pairs created by rendering a virtual 3D laboratory environment. The environment contains objects located at various depths, resulting in a large range of disparities. Each image has size 640-by-480 pixels.

We simulated the effect of eye movements by extracting sub-windows from a single pair of stereo images, where the locations of the extracted sub-windows changes over time. In particular, we define fixation points in the left and right images. The left and right fixation points share the same vertical position, but are offset horizontally by an amount modeling the vergence angle. If the vergence angle is equal to the disparity in the original image, then the fixation points correspond to the same point in the virtual environment.

The stereo input to the saccade control mechanism is obtained by extracting the largest equally sized sub-windows from the left and right images such that the left and right fixation points are aligned in the two sub-windows. Let *M* and *N* denote the vertical and horizontal sizes of the images in pixels. If the horizontal offset between the left and right fixation points is *d*, then the upper left and lower right locations of the sub-windows are (*d*, 1) and (*M*, *N*) in the left image and (1,1) and (*M*-*d* + 1, *N*) in the right image. These sub-windows are down-sampled by a factor of 4, resulting in a coarse scale representation. We applied bicubic interpolation to implement the image down-sampling.

The stereo input to the vergence control mechanism is obtained by extracting pairs of square sub-windows centered at the fixation points and then possibly down-sampling to generate pairs of 55-by-55 pixel images corresponding to three scales: coarse, medium, and fine. The coarse scale input is obtained by down-sampling 220-by-220 pixel sub-windows by a factor of 4. The medium scale input is obtained by down-sampling 110-by-110 pixel sub-window by a factor of 2. The fine scale input is obtained by extracting 55-by-55 pixel sub-windows without down-sampling.

Simulations consist of 10 frame periods of fixation separated by saccades. Assuming a frame rate of 25 frames per second, each fixation lasts for 400 ms. During each fixation, the horizontal location of the right fixation point is adjusted according to the command given by the vergence control mechanism. If learned correctly, the vergence control mechanism adjusts the horizontal shift so that both sub-images are centered on the same point in the scene. We define the retinal disparity to be the difference between the shift and the original image disparity. When the retinal disparity is 0, the images in the left and right sub-windows are aligned. The binocular image pair is changed after every 30 fixations (300 frames), modeling a change in the scene.

Between fixations, the system uses the saccade mechanism to choose the left image fixation point. The right image fixation point is located at the same vertical location but is offset horizontally by a shift, which models the vergence angle. The initial vergence angle of each fixation is the same as the last vergence angle from the previous fixation.

The iCub environment runs in the iCub simulation platform (Tikhanoff et al., 2008). The iCub is a humanoid robot with an active binocular vision system. The horizontal and vertical field of view are 64° and 50°, respectively. To simplify the simulation of the environment, we created the iCub world using some simple objects as shown in Figure 1. The virtual environment in front of the iCub robot contains a number of frontoparallel planar surfaces: a large background plane at a depth of 2 m, and five smaller planes of size 0.6 m × 0.6 m square placed at varying depths between the iCub and the background plane and at varying frontoparallel offsets. The planes are textured with images randomly chosen from the McGill natural image database (Olmos and Kingdom, 2004). Binocular images pairs with size 320-by-240 pixels are generated by rendering this environment based on the positions and gaze angles of the two eyes.

In our simulations, the iCub remains stationary, except for changes in the gaze directions of its left and right eyes, which are controlled by three degrees of freedom: the version, tilt, and vergence angles. As in the Tsukuba environment, simulations consist of 10 frame periods of fixation separated by saccades. However, the left and right fixation points are both fixed at the center of the images. Saccades between fixations are implemented by changing the version and tilt angles, which are common to both eyes. During saccades, the vergence angle remains constant. During fixation, vergence eye movements are implemented by changing the vergence angle between the two eyes while keeping the version and tilt angles fixed. The iCub environment is more realistic than the Tsukuba environment, but simulations are more time consuming due to the rendering. Every 30 fixations (300 frames), the virtual environment is changed by choosing a new set of images from the database to apply to the planar surfaces and by randomizing the depths and positions of the smaller surfaces.

### Perceptual Representation Mechanism

The vergence and saccade control mechanisms are based on the same perceptual representation mechanism applied to the left and right eye inputs. The left and right eye images are divided into 2D arrays of 10-by-10 pixel patches. For saccade generation, the patches are offset by a stride of one pixel. For the vergence control, the patches are offset by a stride of five pixels. At each scale *s* and for each pair *j* of corresponding patches in the left and right eye sub-windows, we concatenate the image intensities into a 200-dimensional binocular vector,

(1) $${\text{x}}_{s,j}=\left[\begin{array}{c} {\text{x}}_{\text{L},s,j}\\ {\text{x}}_{\text{R},s,j} \end{array}\right]\in R^{200}$$

where *s* ∈ {C, M, F} (C, M, F stand for the three scales: coarse, medium, and fine scale, respectively), and the monocular vectors, ***x***_L,_*_s,j_* and ***x***_R,_*_s,j_*, contain the pixel intensities from the left and right image patches, which are normalized separately to have zero mean and unit variance.

The representation mechanism has three sets of *N* = 324 binocular feature extractors, each set corresponding to one scale. The binocular stimulus ***x****_s_*_,_*_j_* is encoded by the set of feature extractors at the associated scale *s*. The *n-*th feature extractor in scale *s* is defined by a two dimensional subspace of the input space, which is spanned by the basis defined by the columns of a matrix $\Phi_{s,n}\;\in \;R^{200\times 2}$ where *n* ∈ {1, … , *N*}. Given an input patch vector ***x****_s_*_,_*_j_*, the response of the *n*-th feature extractor is defined to be the squared length of the projection of ***x****_s_*_,_*_j_* onto the subspace defined by Φ*_s,n_*:

(2) $$r_{n}\left({\text{x}}_{s,j}\right)={∥\Phi_{s,n}^{T}{\text{x}}_{s,j}∥}^{2}$$

where the superscript *T* denotes the transpose operation. For each feature extractor Φ*_s,n_*, we define the response map to be the 2D set of responses of that feature detector to the 2D array of patches.

The operations involved in computing the response maps are similar to those used in computing the binocular energy model, which is commonly used to model the responses of orientation, scale, and disparity tuned binocular complex cells in the primary visual cortex (Ohzawa et al., 1990). The subspace projection operation computes two weighted sums of the binocular image intensities. Thus, each basis vector (column of Φ*_s,n_*) is analogous to the linear spatial receptive field of a binocular simple cell. After learning, these basis vectors exhibit Gabor-like structures and are in approximate spatial phase quadrature (Chandrapala and Shi, 2014). As in the binocular energy model, the response *r_n_*(***x****_s,j_*) combines the squared magnitudes of two binocular simple cells. The magnitude of the response reflects the similarity between the binocular image patch and the binocular receptive fields.

The subspaces are initialized randomly and develop according to the update rules for the GASSOM algorithm described in Chandrapala and Shi (2014). The GASSOM exploits the concept of sparsity by using only one subspace to represent the input and captures the slowness by assuming that the subspace representing ***x***(*t*) is more likely to be the same as the one that generated ***x***(*t* − 1). The model parameters, e.g., the matrices Φ*_s,n_*, are learned in an unsupervised manner, by maximizing the likelihood of the observed data. The update to each subspace is calculated by

(3) $$\Delta \Phi_{s,n}=\underset{j}{\sum }h_{s,n}\cdot {\tilde{\text{x}}}_{s,j,n}\cdot \frac{{\text{x}}_{s,j}^{T}\Phi_{s,n}}{∥{\hat{\text{x}}}_{s,j,n}∥∥{\text{x}}_{s,j}∥}$$

where $h_{s,n}$ determines the amount that subspace $\Phi_{s,n}$ is updated towards the observation, ${\tilde{\text{x}}}_{s,j,n}\;=\;{\text{x}}_{j}-{\hat{\text{x}}}_{s,j,n}$ is the difference between the input ***x****_j_* and its projection onto subspace Φ*_s,n_*, where the projection is computed by ${\hat{\text{x}}}_{s,j,n}=\Phi_{s,n}\Phi_{s,n}^{T}{\text{x}}_{j}$. Thus, each subspace at time *t* is updated by

(4) $$\Phi_{s,n}\left(t\right)=\Phi_{s,n}\left(t-1\right)+\lambda \Delta \Phi_{s,n}\left(t-1\right)$$

where λ > 0 is the learning rate.

Under the AEC framework, both the GASSOM model parameters and the parameters of the vergence control policy (described below) are learned simultaneously.

### Vergence Control Mechanism

The vergence control mechanism maps the visual input to a probability distribution over a discrete set of 11 possible vergence actions *A*_verg_. For the Tsukuba environment, the 11 discrete vergence actions are *A*_verg_ = {−16, −8, −4, −2, −1, 0, 1, 2, 4, 8, 16} pixels, which modify the shift between the centers of the left and right sub-windows. For the iCub environment, the vergence actions are *A*_verg_ = {−3.2, −1.6, −0.8, −0.4, −0.2, 0, 0.2, 0.4, 0.8, 1.6, 3.2} degrees.

The vergence control policy is implemented by a two layer neural network. The input to the network is a 3*N*-dimensional vector:

(5) $$r_{\text{verg}}=\left[\begin{array}{c} r_{\text{C}}\\ r_{\text{M}}\\ r_{\text{F}} \end{array}\right]$$

where for each *s* ∈ {C, M, F}, ${\text{r}}_{s}\;\in \;R^{N}$ is obtained by spatially pooling the response maps of the feature detectors at scale *s*:

(6) $${\text{r}}_{s}=\left[\begin{array}{c} \frac{1}{P}\overset{P}{\underset{j=1}{\sum }}r_{1}\left({\text{x}}_{s,j}\right)\\ \vdots \\ \frac{1}{P}\overset{P}{\underset{j=1}{\sum }}r_{N}\left({\text{x}}_{s,j}\right) \end{array}\right]$$

where *P* = 100 is the number of the patches.

The output layer of the network contains 11 neurons, each corresponding to a possible vergence command. The vector of activations to the output neurons, ${\text{z}}_{\text{verg}}\in R^{11}$, is computed as:

(7) $${\text{z}}_{\text{verg}}=\theta^{T}{\text{r}}_{\text{verg}}$$

where $\theta^{T}\in R^{972\times 11}$ is a matrix of synaptic weights.

The vector of probabilities for selecting the different vergence actions, $\pi_{\text{verg}}\in R^{11}$, is calculated by applying a softmax operation to the activation vector **z**_verg_:

(8) $$\pi_{\text{verg}}=\mathrm{softmax}\left({\text{z}}_{\text{verg}}∕\beta_{\text{verg}}\right)$$

where the softmax function **y** = softmax(**z**) is defined component-wise by:

(9) $$y_{i}=\frac{\exp\left(z_{i}\right)}{\sum_{k=1}^{11}\exp\left(z_{k}\right)}$$

for *i* ∈{1, … , 11} The temperature parameter, β_verg_, balances exploration and exploitation during reinforcement learning. In the following experiments, β_verg_ is set to 1.

The neural network weights develop according to the natural actor-critic reinforcement learning algorithm (Bhatnagar et al., 2009). In our system, the reinforcement learner seeks a vergence control policy that minimizes the error in the perceptual representation of the sensory input, or equivalently maximizes the fidelity of the perceptual representation. We define the instantaneous reward to be the negative of the average squared reconstruction error across the three scales, which is defined by

(10) $$R_{\text{verg}}=-E_{\text{avg}}=-\frac{1}{3}\underset{s\in S}{\sum }E_{s}$$

where *E_s_* is the mean squared reconstruction error at scale *s* averaged across all patches. The reconstruction error of each patch is defined as the squared length of the residual between the input vector ***x****_s,j_* and its projection onto the best-fitting subspace:

(11) $$E_{s}=\frac{1}{P}\overset{P}{\underset{j=1}{\sum }}{∥{\text{x}}_{s,j}-\Phi_{s,m_{s,j}}^{T}\cdot {\text{x}}_{s,j}∥}^{2}$$

where *m_s,j_* is the index of the best-fitting subspace for ***x****_s,j_*,

(12) $$m_{s,j}=\underset{n}{\text{arg max}}\;{∥\Phi_{s,n}^{T}\cdot {\text{x}}_{s,j}∥}^{2}.$$

The weight matrices of the value and policy networks are updated during fixation, but not across saccades.

### Saccade Control Mechanism

#### Binocular Saliency Model

**Figure 3:**
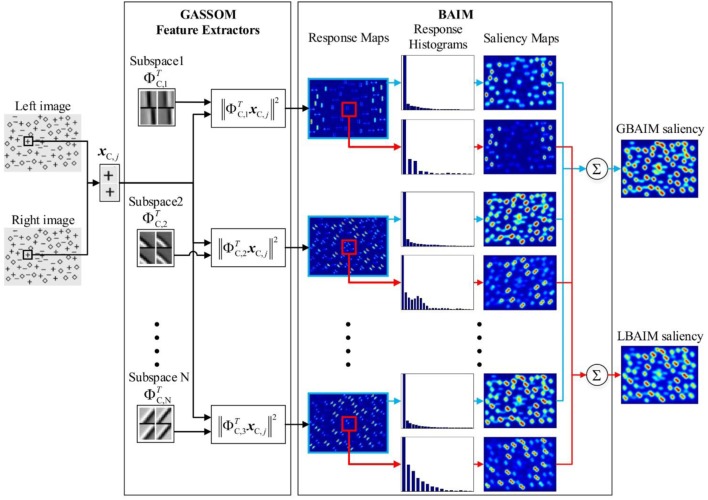
The architecture of the binocular attention based on information maximization (BAIM). The left and right parts of image patches and basis vectors are shown as 10-by-10 pixel images and aligned vertically. Red regions in the response maps and saliency maps correspond to high values while blue regions correspond to low values. The red squares in the response maps represent the local area where response histograms are generated.

The binocular saliency map is generated using binocular attention based on information maximization (BAIM), which we propose as a binocular extension of the AIM model of Bruce and Tsotsos (2009). The BAIM architecture is illustrated in Figure 3. In essence, we replace the monocular basis functions learned by ICA in the AIM model with the binocular basis functions learned by GASSOM. Whereas the monocular basis functions encode only texture information, the binocular basis functions used here jointly encode depth and texture information. Since our experiment use only gray scale images, we do not jointly encode color as in the experiments by Bruce and Tsotsos (2009). However, the extension to include color is straightforward, involving only an expansion of the size of the input vector.

The computations to obtain the salience map are performed at the coarse scale. We assign saliency values to 10-by-10 pixel patches, which are extracted with a stride of one. Given coarse scale images with size *M*_1_ × *M*_2_, we obtain a 2D array of $P_{\text{sal}}=\left(M_{1}-9\right)\times \left(M_{2}-9\right)$ binocular patches. To obtain a saliency map at the original resolution, we use zero padding to increase the size of the coarse scale map to *M*_1_ × *M*_2_ pixels and then upsample by a factor of 4.

The saliency value for the *j-*th coarse scale binocular image patch, *S*(***x****_C,j_*), is a measure of how informative or unlikely the responses of the GASSOM feature detectors are in the context of the responses from the other patches. More specifically, it is the sum of saliency values computed for individual feature extractors in the GASSOM representation, *S_n_*(***x****_C,j_*):

(13) $$S\left({\text{x}}_{\text{C},j}\right)=\sum_{n=1}^{N}S_{n}\left({\text{x}}_{\text{C},j}\right).$$

The saliency of each feature extractor is the Shannon self-information of the response:

(14) $$S_{n}\left({\text{x}}_{\text{C},j}\right)=-\text{ln }p_{n}\left[r_{\text{C},n}\left(x_{\text{C},j}\right)\right]$$

where *p_n_*[⋅] is the probability distribution of the responses of the *n*-th feature extractor at the coarse scale, which we estimate empirically using a histogram. Each response map is normalized to the range 0–1. The histogram of each response map is generated using $K=\sqrt{P_{\text{sal}}}$ equal width bins:

(15) $$p_{n}\left[r_{\text{C},n}\left({\text{x}}_{\text{C},j}\right)\right]=\alpha +\left(1-\alpha \right)\overset{K-1}{\underset{k=0}{\sum }}h_{n}\left(k\right)\cdot {\text{1}}_{\left[\frac{k}{K},\frac{k+1}{K}\right)}\left(r_{\text{C},n}\left({\text{x}}_{\text{C},j}\right)\right)$$

where

(16) $${\text{1}}_{\left[a,b\right)}\left(x\right)=\left\{\begin{array}{cc} 1 & \text{ if}x\in \left[a,b\right)\\ 0 & \text{ if}x\notin \left[a,b\right) \end{array}\right.$$

is an indicator function with *a* and *b* as free parameters. The parameter α = 10^−6^ is a small number that guarantees that the response probabilities are non-zero. The coefficients

(17) $$h_{n}\left(k\right)\;=\;\frac{1}{P_{\text{sal}}}\overset{P_{\text{sal}}}{\underset{j=1}{\sum }}{\text{1}}_{\left[\frac{k}{K},\frac{k+1}{K}\right)}\;\left(r_{n}\left({\text{x}}_{C,j}\right)\right)\text{ for }k\in \left\{0,1,2,...,K-1\right\}$$

are empirical estimates of the probability that the response falls into the *k*-th bin computed over $P_{\text{sal}}$ patches.

We considered both global binocular attention based on information maximization (GBAIM) and local binocular attention based on information maximization (LBAIM) versions of the saliency map, which differed according to patches used to estimate the coefficients *h_n_*(*k*) in Eq. 17. In the GBAIM model, the coefficients were computed by summing over all coarse scale patches. In the LBAIM model, the sum was over only the coefficients from a 31 × 31 array of patches centered around the current fixation point. The LBAIM model tends to favor patches where the GASSOM responses are more unlike those in the local neighborhood of the current fixation point.

**Figure 4:**
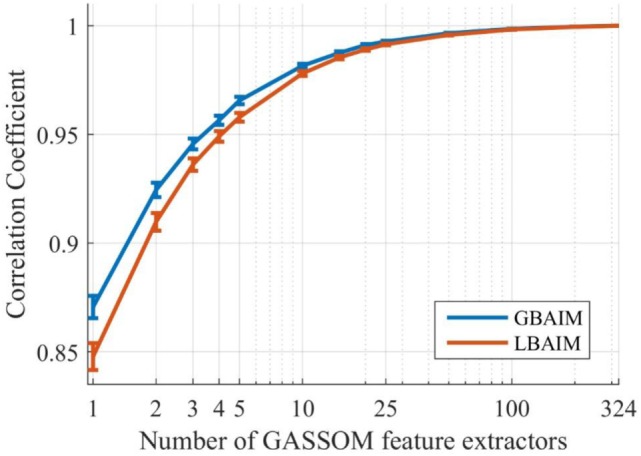
The average correlation coefficient (CC) values between the binocular attention based on information maximization (BAIM) saliency maps generated by all feature extractors and the saliency maps generated by different subsets of feature extractors. A logarithmic scale is used on the *x*-axis. Error bars represent 95% confidence intervals for the mean values of the CCs.

To speed up computations, we use only a random subset of the GASSOM feature extractors to compute the sum in Eq. 13. To determine the size of the subset, we computed the correlation coefficients (CCs) to measure the similarity between the saliency maps generated by random subsets of feature extractors and by all feature extractors. The CC between two saliency maps *S*_1_ and *S*_2_ is defined as:

(18) $$\text{CC}=\frac{\sum_{j}\left(S_{1}\left({\text{x}}_{\text{C},j}\right)-\mu_{1}\right)\left(S_{2}\left({\text{x}}_{\text{C},j}\right)-\mu_{2}\right)}{\sqrt{\sum_{j}{\left(S_{1}\left({\text{x}}_{\text{C},j}\right)-\mu_{1}\right)}^{2}}\sqrt{\sum_{j}{\left(S_{2}\left({\text{x}}_{\text{C},j}\right)-\mu_{2}\right)}^{2}}}$$

where μ_1_ and μ_2_ are the mean saliencies in the maps. Figure 4 plots the CCs averaged over salience maps computed from 1,800 pairs of binocular images from the Tsukuba dataset. For each saliency map, the subsets of a certain number of feature extractors are chosen randomly from all 324 feature extractors. Using only 25 feature, extractors generates a BAIM saliency map that is very similar to the one using all 324 features. Thus, in our experiments, we sum over 25 randomly selected feature extractors.

#### Inhibition of Return

Given the current fixation, a saccade target for the next fixation is generated by combining the saliency map at the full image resolution with a simple IOR mechanism (Dorris et al., 2002), which prevents the system from saccading to recently visited image locations.

Defining the full resolution saliency map by *S*(*j*) where *j* indexes the patch, we choose the next fixation point by sampling from the probability distribution

(19) $$p\left(j\right)=\frac{S\left(j\right)\cdot \text{IOR}\left(j\right)}{\sum_{k}S\left(k\right)\cdot \text{IOR}\left(k\right)}$$

where IOR(⋅) is a mask that suppresses recently visited image locations.

Posner and Cohen (1984) indicate that the currently attended region is inhibited for approximately 500–1,000 ms. Since fixations last for 10 frames, and assuming a frame rate of 25 frames per second, we prevent the system from visiting the last two fixation points, resulting in a 800 ms long IOR window. Assuming the indices of most recent and second most recent fixation locations are *j*_1_ and *j*_2_, we set

(20) $$\text{IOR}\left(j\right)=f\left(j,j_{1},\sigma_{1}^{2}\right)\cdot f\left(j,j_{2},\sigma_{2}^{2}\right)$$

where

(21) $$f\left(j,k,\sigma^{2}\right)=1-\exp\left(-\frac{{∥{\text{p}}_{j}-{\text{p}}_{k}∥}^{2}}{2\sigma^{2}}\right).$$

where **p***_j_* is the 2D image location of patch *j*, σ_1_ = 20 pixels and σ_2_ = 10 pixels.

## Results

### BAIM-Driven Saccades Accelerate Vergence Learning

We compared the rate at which vergence control policies emerged and the quality of the final polices under different saccade control policies including a random policy where *p*(*m, n*) in Eq. 19 was uniform over all image locations and policies where *S*(*m, n*) in Eq. 19 was computed according to the saliency model of Itti et al. (1998), the AIM model (Bruce and Tsotsos, 2009), and the GBAIM and LBAIM models proposed here.^2^

**Figure 5:**
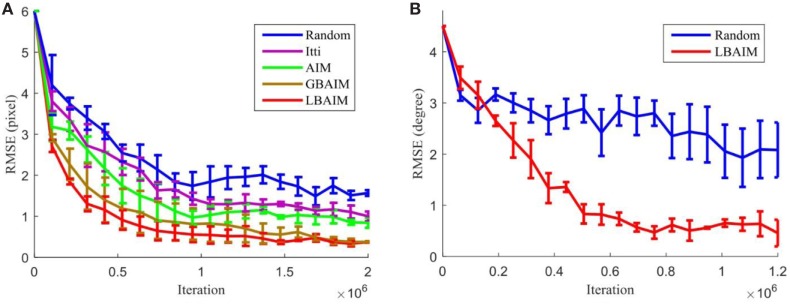
The evolution of the root mean squared error (RMSE) of the vergence control policy for different saccade control policies over training in **(A)** the Tsukuba environment and **(B)** the iCub environment. Error bars indicate the SD computed over three training runs.

Figure 5 shows the evolution of the root mean squared error (RMSE) between the learned vergence control policies during training and the ideal policy that zeros out the input disparity in both the Tsukuba and the iCub environments. For the Tsukuba environment, we ran three training trials for each saccade control policy. We set the same learning rate for the vergence control learner to make all the saccade methods comparable. Each trial used binocular inputs generated by disjoint sets of 120 randomly chosen stereo images, but we used the same image sets to train the different saccade policies. We sampled the vergence policies at 20 equally spaced checkpoints during training. At each checkpoint, we presented the policy with inputs with initial disparities ranging from −20 to +20 pixels and let the vergence policy run for 10 iterations. The RMSE in pixels was computed as the square root of the mean squared retinal disparity after 10 iterations averaged over all initial disparities and 100 inputs per disparity. The images used to characterize the policies at all testing points and for all saccade policies were identical and disjoint from those used in training. For the iCub environment, we also ran three training trials for each saccade control policy. Each trial was conducted in a different randomly generated environment with disjoint sets of image textures mapped on to the surfaces. The same environments were used to train under different saccade policies. During testing, the iCub was presented with frontoparallel surfaces at depths ranging from 0.5 to 2.0 m and textures disjoint from those used in training and allowed to verge for 10 iterations starting from initial vergence angles ranging from 0° to 10°. The RMSE of the final vergence angle was averaged over all depths and all initial vergence angles.

As shown in Figure 5, the random saccade policy performs the worst, resulting in the largest vergence control policy RMSE at the end of training. The two BAIM-driven saccade policies result in the best final performance. Although there is little difference between the two final policies, the LBAIM model exhibits faster vergence learning, with a faster decrease in the RMSE. The two monocular saccade models result in vergence control policies with RMSE values lying between those learned under random and binocularly driven saccades, with the AIM model exhibiting slightly faster learning and lower final RMSE.

**Figure 6:**
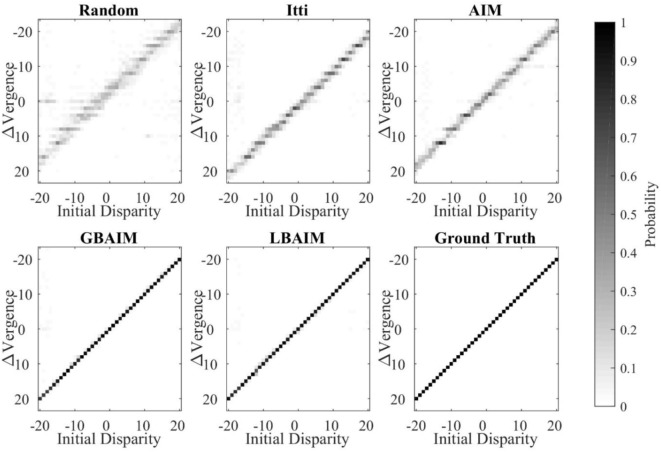
Visualizations of the final vergence policies after training. Each policy is presented as an image. The horizontal axis indicates the initial disparity and the vertical axis indicates the change in vergence after 10 iterations of the policy. The intensity of each pixel corresponds to the probability of the change in vergence given the initial disparity, i.e., the entries in each column sum to 1. For the ground truth policy, the change in vergence is always the negative of the initial disparity.

Figure 6 shows visualizations of the final policies learned in the Tsukuba environment. It is clear that the final vergence control policies learned using the BAIM-driven saccades are closer to the ground truth. The “blurred” images for the policy learned using random and monocular saliency driven saccades indicate that the policies are less reliable in zeroing out the retinal disparity.

### BAIM-Driven Saccades Select Image Regions with Higher Entropy

To understand better how the different saccade policies lead to vergence control policies with different performance, we examined the entropy of the image regions around the fixation points chosen by the different saccade policies. The entropy is defined as:

(22) $$E=-\underset{i}{\sum }p_{i}\log_{2}\left(p_{i}\right)$$

where *p_i_* is the probability of the pixel intensity value *i*, which is estimated from the histogram of pixel intensity values. The entropy is a measure of the spread of gray values and is one measure of the information content. Image regions whose pixels all have the same intensity have zero entropy. Textured regions will have more variability in gray levels, and therefore a higher entropy. Intuitively, it will be harder to learn vergence from regions without texture (with lower entropy).

**Figure 7:**
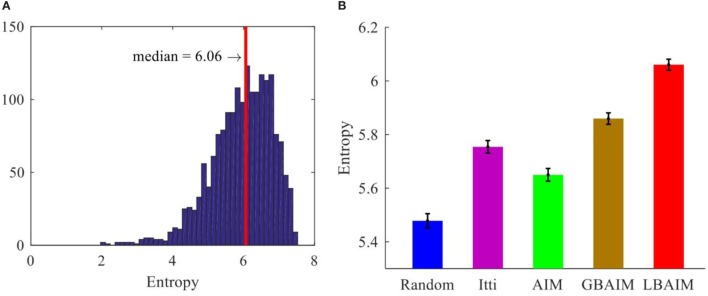
**(A)** The entropy histogram of the fine scale sub-windows selected by local binocular attention based on information maximization (LBAIM). The red line indicates the median entropy. **(B)** The median entropy of the patches at the fixation points selected by different saccade control methods. The error bars represent the SEM.

Figure 7 shows the median entropies computed over the 55-by-55 pixel fine scale sub-windows at the fixation points selected by the different saccade control methods. The statistics were collected over 1,800 sub-window pairs. For each of the 180 stereo image pairs obtained by taking every 10th frame from the 1,800 stereo image pairs in the Tsukuba dataset, we ran the saccade/vergence policy learned after 100,000 iterations (the first checkpoint in Figure 5) for 10 fixations (100 frames), and averaged the entropies of the left and right sub-windows. Our testing results for the learned systems at other checkpoints were similar.

### BAIM-Driven Saccades Lead to Improved Encoding of Small Disparities

By selecting different fixation points, the different saccade policies expose the perceptual representation to binocular patches with different statistics. Since the feature extractors evolve to maximize the likelihood of the observed data, these differences in the input statistics will be reflected as differences in the learned feature extractors.

To study these differences, we learned feature detectors in the Tsukuba environment using sub-windows centered at fixation points chosen by the different saccade control policies. Since differences in the vergence control policies will affect the input disparity statistics, we chose the vergence angles so that the retinal disparities *d* between the fixation points followed a discrete truncated Laplacian distribution between −40 and +40 pixels:

(23) $$P\left(d\right)=Me^{{\;}^{-\left|d\right|}/{}_{D}},\;d\in \left\{-40,-39,...,40\right\}$$

where *M* is a normalization factor that ensures that *P*(*d*) sums to one. The parameter *D* controls the spread of the input disparities. This enabled us to isolate the effects of the saccade policies on the perceptual representations.

**Figure 8:**
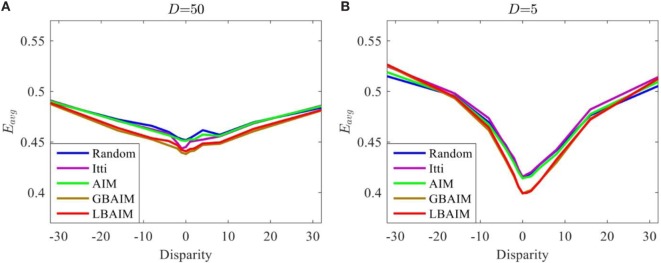
The average reconstruction error for the perceptual representations learned under different saccade control policies plotted as a function of the retinal disparity between the sub-windows. The retinal disparity at the fixation points followed truncated Laplacian distributions with parameters: **(A)** *D* = 50 and **(B)** *D* = 5.

Figure 8 shows the average reconstruction error *E*_avg_ of the perceptual representations after training under different saccade control policies and assuming two different disparity statistics. The values of *E*_avg_ are plotted as a function of the retinal disparity at the fixation points. They are computed by averaging the values of *E*_avg_ computed according to Eq. 10 over sub-windows taken at 1,000 fixation points from 100 images in the Tsukuba dataset (10 fixations per image). Images used in testing were disjoint from those used in training.

In general, the curves have a characteristic “V” shape, being symmetric around and achieving their minima at zero disparity. This suggests that the perceptual representation is adapted to best represent binocular stimuli with zero disparity and that there are more feature extractors tuned to zero disparity. There is no preference for positive or negative disparity stimuli. These observations are consistent with the distribution of retinal disparities in the input, which is peaked at and symmetric around 0. The “V” shape indicates small reconstruction error at 0 and large average reconstruction error at large disparities. The “V” shape is more pronounced the more tightly the input disparity statistics are clustered around zero disparity, which is obtained by choosing a smaller value of *D* in Eq. 23.

**Figure 9:**
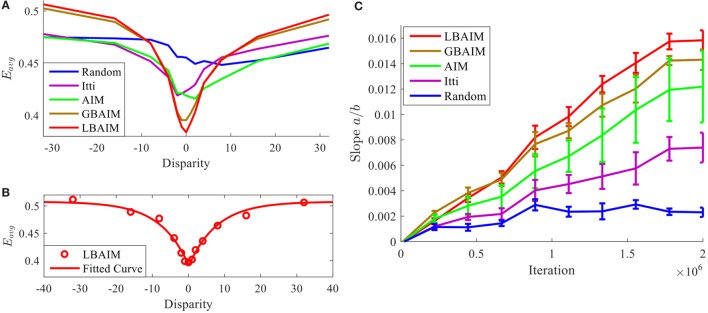
**(A)** The average reconstruction error as a function of the retinal disparity. **(B)** The experimentally estimated reconstruction errors of local binocular attention based on information maximization (LBAIM) and the corresponding curve fit. **(C)** The change of the slope *a*/*b* over the training process. Larger slopes indicate that the perceptual encoding exhibits a stronger preference for inputs with zero disparity. Error bars represent the SD computed over three training runs.

Figure 9A shows the reconstruction error curves of the perceptual representations learned after joint learning under different saccade control policies. The differences are much more pronounced, with curves corresponding to the BAIM-driven saccade policies exhibiting much more pronounced “V” shapes. To quantify how pronounced those “V” shapes are, we fit the reconstruction error curves with the function

(24) $$f_{a,\mu ,b,c}\left(d\right)=c-a\cdot \;\text{exp}\;\left(-\frac{\left|d-\mu \right|}{b}\right)$$

where *c* is a vertical offset, μ sets the location of the minimum, and *a* and *b* control the slope and depth of the “V,” respectively. Figure 9B shows one example of a fit. We measure the sharpness of the “V” by the ratio *a*/*b*, which is the absolute value of the slope at μ. Figure 9C shows the evolution of the slope during training under the different saccade control policies. In all cases, the slope increases over time, indicating that the basis functions evolve so that they provide a better encoding of stimuli with zero disparity. However, the rate and magnitude at which the slope increases are largest under the BAIM-driven saccade control policies.

### LBAIM-Driven Saccades Target Locations with Different Disparities

Figure 5 indicates that the RMSE of the final vergence control policies learned under the GBAIM and LBAIM saccade policies are similar, but that the vergence control policy emerges faster under LBAIM. The similarities between the reconstruction error curves and slope trajectories of the perceptual representations learned under GBAIM and LBAIM in Figures 8 and 9 suggest that the faster learning is not due to differences in the perceptual representation. Rather, we suggest the learning is faster because LBAIM presents more challenging vergence control stimuli to the system.

**Figure 10:**
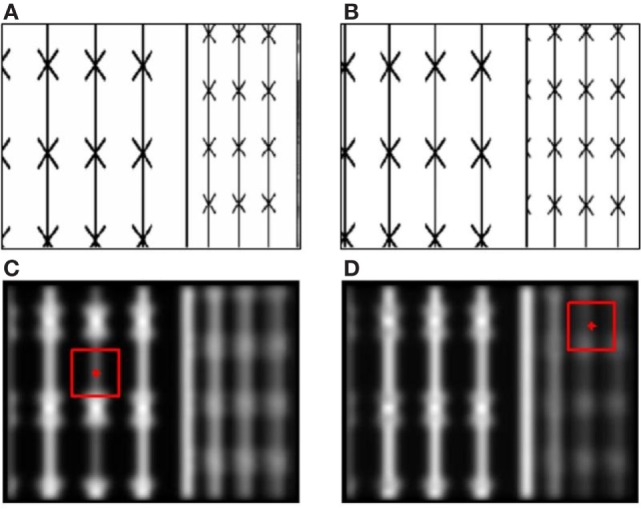
**(A,B)** Left **(A)** and right **(B)** eye images obtained from the iCub simulator when the robot was viewing two planar objects: one on the left which is closer and the other on the right which is farther. **(C,D)** Examples of the local binocular attention based on information maximization (LBAIM) saliency map computed with the iCub fixating on the closer **(C)** and farther **(D)** objects. Bright regions correspond to high saliency. The red points indicate the fixation point. The red squares indicate the local area over which the empirical response histogram is computed.

As a concrete example, Figure 10 shows two examples of saliency maps computed by LBAIM in the iCub environment. The maps were computed in the same environment, which had two objects with the same textures in front of the iCub: one on the left at a closer distance and one on the right at a farther distance and partially occluded by the closer object. Figures 10A,B show example images from the left and right eye cameras. The two saliency maps were computed assuming the iCub was fixating either on the closer or the farther object. Comparing their intensities, we observe that points on the closer object become more salient when the iCub is fixating on the farther object and *vice versa*.

For a more comprehensive and quantitative comparison, we estimated the expected absolute disparity difference between current and next fixations for different saliency models according to

(25) $$\bar{\Delta D}=\frac{1}{P}\sum_{i,j}\left|D_{i}-D_{j}\right|p\left(i|j\right)$$

where *i* indexes all possible next fixations, *j* indexes the current fixation point, *D_i_* is the disparity at *i*, and *P* is the number of current fixation points considered. The term

(26) $$p\left(i|j\right)=\frac{S\left(i|j\right)}{\sum_{k}S\left(k|j\right)}$$

is the probability of choosing the next fixation point *i* given the current fixation point *j*. It is similar to Eq. 19 except that we do not include the IOR and that we make explicit the dependency of the saliency map *S*(*i*|*j*) on the current fixation. For all of the saliency mechanisms except LBAIM, the saliency map does not change with the current fixation location.

**Figure 11:**
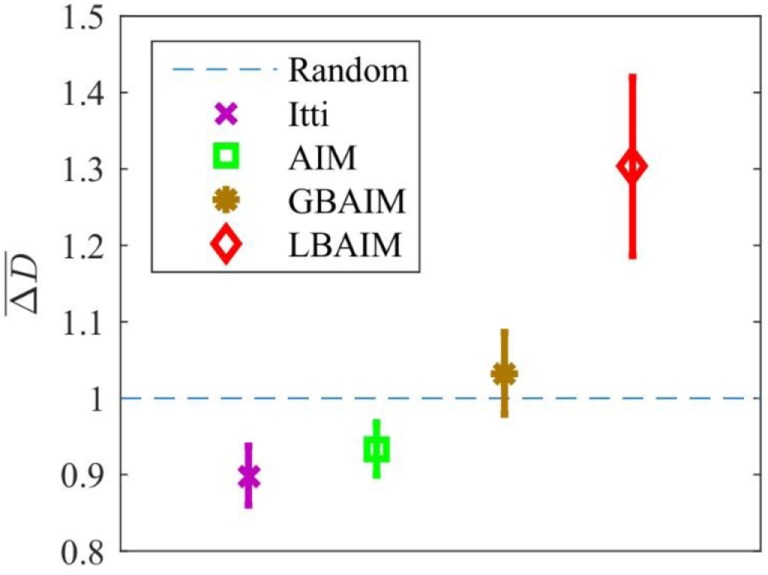
The estimated expected absolute disparity difference, $\bar{\Delta D}$, for saccades generated by the different saliency models normalized by the expected difference estimated using random saccades. The error bar represents the SD.

The LBAIM saliency model shows a clear preference toward selecting targets with disparities that are different from that at the current fixation point. Figure 11 shows the expected absolute disparity difference under different saccade policies normalized by the expected absolute disparity difference under the random saccade policy. The expected differences were estimated from data in the Tsukuba data set, for which ground truth disparity data are available. We selected 200 binocular images randomly from the 1,800 image frames in the video of the Tsukuba dataset. For each binocular image, we computed the saliency maps at 10 fixation points (i.e., *P* = 2,000).

### Change of Reconstruction Error within One Fixation

The average reconstruction error of the perceptual representation, *E*_avg_, plays a critical role in this system. Both the learning of the perceptual representation and the learning of the vergence control policy seek to minimize *E*_avg_. Large decreases in *E*_avg_ during fixation suggest that the vergence control reinforcement learning is being exposed to “challenging” situations where there is potential for large changes in the reward.

**Figure 12:**
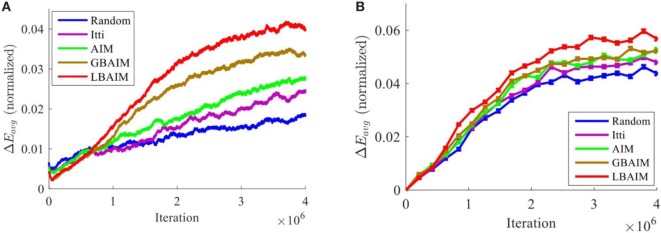
**(A)** The running average of the normalized decrease in the average reconstruction error during fixation increases during training. **(B)** To isolate the effect of the saccade policy from differences in the perceptual representation and the vergence policy, we plot the normalized decrease in average reconstruction error when the same perceptual representation and vergence policy were used.

Figure 12A shows how the average decrease in *E*_avg_ during fixation evolves over training under the different saccade control policies. We define the normalized decrease across one fixation (10 frames) as the difference between the values of *E*_avg_ at the start and end of a fixation normalized by the value of *E*_avg_ at the start of fixation, and compute the running average across 3,000 fixations. The normalized decreases in *E*_avg_ under the BAIM-driven saccade policies exhibit much larger and faster increases across training than the monocular saliency and random policies.

We isolated the effect of the choice of saccade target on the average normalized decrease in *E*_avg_ by using the same perceptual representation and vergence policies during fixation, but choosing the initial fixation points according to the different saccade control policies. In the results reported here, we used the perceptual representation and vergence policies learned by LBAIM, but other choices gave similar results. Figure 12B shows that the ordering of the policies is preserved, but the relative differences between the curves for different saccade policies are smaller.

## Discussion

We have described an integrated active vision system that combines vergence control learning with saccade control based on two novel binocular saliency models: LBAIM and GBAIM. These models are based on binocular feature extractors that simultaneously encode both texture and depth information and that are computed in a way similar to the binocular energy model, a common computational model for disparity and orientation selective cells in the primary visual cortex. The algorithm assigns high saliency to regions with high information content considering either the LBAIM or GBAIM context.

Similar to the development of human infants, both the saccadic and vergence control policies in our model are immature at the start of simulation and emerge through interaction with the environment. For the saccade policy, this is because the basis functions are randomly initialized. For the vergence policy, this is because both the basis functions and the weights in the actor and critic networks are randomly initialized. The order in which these policies emerge is the same as in humans. In our simulation, the saccade policy develops before the vergence policy. In humans, infants begin to look at edges or places with sharp and high-contrast features from 1 to 2 months of age (Bronson, 1990, 1991; Colombo, 2001). Vergence develops at about 4 months of age (Aslin, 1977; Hainline and Riddell, 1995). However, the absolute time scale at which these behaviors emerge differs. We measure the time it takes these policies to emerge in terms of fixations, since the number of iterations required by the model is somewhat arbitrary, as it depends upon the choice of the time step. In our simulations, the vergence policies when following the LBAIM saccade policy emerge after about 30,000 fixations (300,000 iterations) (Figure 5). Adult humans execute around 10^5^ fixations/day,^3^ so this corresponds to 3 days for an adult, but we expect this number to be a bit larger as infants spend less time awake and their saccade policies are immature. The saccade policy develops very quickly, after about 100 fixations (see Supplementary Material), but this is largely due to the fact that it depends only on the basis functions, as much of the processing is hard coded in our model. We expect that if we incorporated learning into more aspects of the saccade control policy as is likely the case in humans, we would obtain a much slower rate of emergence.

We could obtain simulations where vergence policies emerge on the same timescales as in humans by lowering the learning rate. However, we did not do so to avoid excessively long computation time. We are primarily interested in the relative rates, rather than absolute rates at which the policies emerge. Since we use the same learning rate in all simulations, the faster rate at which the vergence policy emerges when following the BAIM saccade policies indicates that the system is actively choosing inputs that allow for the most effective learning. While it is clear that saccades are driven by a number of factors beyond obtaining effective inputs to train vergence control, our model does suggest a complementary, and hitherto largely unappreciated, potential role for saliency-driven saccadic eye movements.

Our experimental results show that vergence control policies learned with saliency-driven saccades all exhibit higher accuracy and are learned faster than when saccades are driven randomly (Figures 5 and 6). The BAIM-driven saccade policies result in the highest accuracy and fastest learning. The primary function of saccades (and attention in general) is commonly thought to direct the limited neural processing of an organism to more important stimuli. Our results suggest a new complementary role of saccades in aiding in the learning of behavior.

Through our experiments with this model, we have identified a number of different interacting factors that account for the improved performance and faster learning.

First, differences in the saccade policies expose the system to input patches with different statistics. All of the attention-based saccade control models direct gaze toward image regions with higher entropy than encountered with randomly generated saccades (Figure 7). Patches selected by the LBAIM models have the highest entropy, followed by the GBAIM, Itti, and AIM models.

Second, since the perceptual representations adapt to the input statistics, these differences lead to differences in the perceptual representations. Higher entropy patches contain more texture, which provides more visual cues to disparity. Perceptual representations learned using higher entropy patches encode differences between zero and non-zero disparities better. Figure 8 shows the dependency between the reconstruction error and the input disparity for the different perceptual representations. The differences between the reconstruction errors for zero and non-zero disparities follow the same trend as the entropy, being the smallest for the random policy, and the largest for the LBAIM and GBAIM policies. The difference also depends upon the statistics of the disparities of the input patches, increasing the more the disparities are concentrated around zero disparity (smaller values of *D* in Eq. 23). The magnitude of the reconstruction error at zero disparity shows the opposite trend, achieving the largest value for the random policy and the smallest values for the BAIM-based policies.

Third, the differences between the reconstruction error curves in Figure 8 are amplified during joint learning by a positive feedback loop setup by the interaction between the learning of the perceptual representation and the learning of vergence control. Both learners seek to maximize the same reward: the average negative reconstruction error of the perceptual representation. Initially, the disparity statistics will be similar, since the vergence policies are initialized with random weights. The different saccade policies will result in slightly different reconstruction error curves. The lower reconstruction error at zero disparity and the larger difference between the reconstruction error at non-zero and zero disparities for the BAIM-driven saccade control will cause the reinforcement learner to favor more strongly the emergence of vergence policies that seek to 0 out the retinal disparity, resulting in a slightly better vergence control policy. In turn, the better vergence policies cause the distribution of retinal disparities presented to the perceptual representation to be more tightly clustered around 0. The perceptual representation will respond by allocating more basis functions to represent zero disparity inputs. This further reduces the reconstruction error for zero disparity inputs and increases the difference at non-zero and zero disparities. This in turn improves vergence control and the cycle continues. Figure 9A shows the net effect of this positive feedback loop by plotting the reconstruction error curves of the perceptual representations learned after joint learning under different saccade control policies. The differences between the perceptual representations are much more pronounced, with curves corresponding to the BAIM-driven saccades policies exhibiting much more pronounced “V” shapes. Figure 9B shows the dynamic evolution of the slope of the “V” shape at its minimum point, which is a measure of the difference in reconstruction error at zero and non-zero disparities. Small initial differences expand rapidly under the positive feedback.

Finally, the BAIM-driven saccades direct the system to focus on more “challenging” situations, i.e., those with larger initial retinal disparity or those where the potential change in the reward are larger. In particular, we find that the LBAIM algorithm, by emphasizing saccade targets that are different from the current fixation, exposes the system to a wider diversity of input patch textures and input disparities, which drives faster learning. Intuitively, saccades between targets at different depths will present more challenges for vergence control, since they require larger change in vergence angle between the two eyes. In our system, vergence angle is preserved across saccades. Thus, saccades to locations with the same absolute disparity as the current fixation will require no change in vergence angle, presenting less of a challenge to the vergence control policy than saccades to targets with a different absolute disparity.

The primary difference between the LBAIM and GBAIM saliency models is that for the LBAIM model, the saliency depends upon the current fixation point, whereas for the GBAIM model, saliency is independent of the current fixation point. This dependency is introduced due to the data used to compute the coefficients of the empirical response histogram (Eq. 17). For the LBAIM model, points whose feature extractor responses are different from those around the current fixation point will be more salient. Since the feature extractors encode disparity, this implies that points with disparity different from the current fixation point will be more salient under LBAIM. Thus, we observe greater differences in disparity between adjacent fixations (Figure 11).

We also observe larger reductions in reconstruction error during fixations (Figure 12). These larger decreases are due to the combination of a number of factors identified earlier. First, the same reduction in retinal disparity will result in a larger decrease in the reconstruction error for the BAIM policies, due to the more pronounced “V” shape of the reconstruction error curves for the BAIM policy. Second, the better quality of the vergence control policies learned under BAIM will result in larger changes in retinal disparity. Third, the choice of saccade targets will influence the change in two ways. By choosing saccade targets with larger entropy, the effect of changes in the retinal disparity on the visual input will be more pronounced, leading to larger changes in *E*_avg_. In addition, choosing initial fixation points with larger retinal disparity will result in larger changes in *E*_avg_. This final factor likely accounts for much of the difference between LBAIM and GBAIM.

Future work will focus on learning all aspects of the saccade and vergence policies simultaneously and under a common parsimonious framework provided by active efficient encoding. For example, although the BAIM saliency maps adapt to the statistics of the sensory input because of changes in the binocular feature detectors learned by the GASSOM algorithm, the way in which the feature detector outputs are integrated to construct the saliency maps is hard coded. We are currently investigating how to learn how to combine feature map outputs to generate saccade policies. In addition, our model of saccades and vergence can be made more realistic. In humans, some of the required vergence change takes place during the saccade (Coubard, 2013), with the remaining disparity canceled by vergence changes after the saccade. Our current model is a simplification of this, since there is no change in vergence during the saccade. It will also be interesting to extend the framework here to include these initial vergence changes by incorporating an estimate of the disparity of the target.

## Author Contributions

All authors contributed to the design of the experiments and the paper writing. QZ conducted the experiments.

## Conflict of Interest Statement

The authors declare that the research was conducted in the absence of any commercial or financial relationships that could be construed as a potential conflict of interest.

## Footnotes

^1^The video is available online at https://youtu.be/axgbhDER1ow.

^2^We implemented Itti’s and AIM saliency model by using the code available at http://www.vision.caltech.edu/harel/share/gbvs.php and http://www-sop.inria.fr/members/Neil.Bruce/#SOURCECODE. Our implementations of the GBAIM and LBAIM are based on the code of the AIM saliency model.

^3^Here, we assume that adult humans are awake for 15 h/day and make 2 saccades/s.
