# Supplementary material for: Joint Learning of Binocularly Driven Saccades and Vergence by Active Efficient Coding
Source: Front Neurorobot. 2017 Nov 3;11:58. doi: 10.3389/fnbot.2017.00058 (PMC5675843; doi:10.3389/fnbot.2017.00058)
Supplement: Supplementary file 1 [file image_1.pdf]

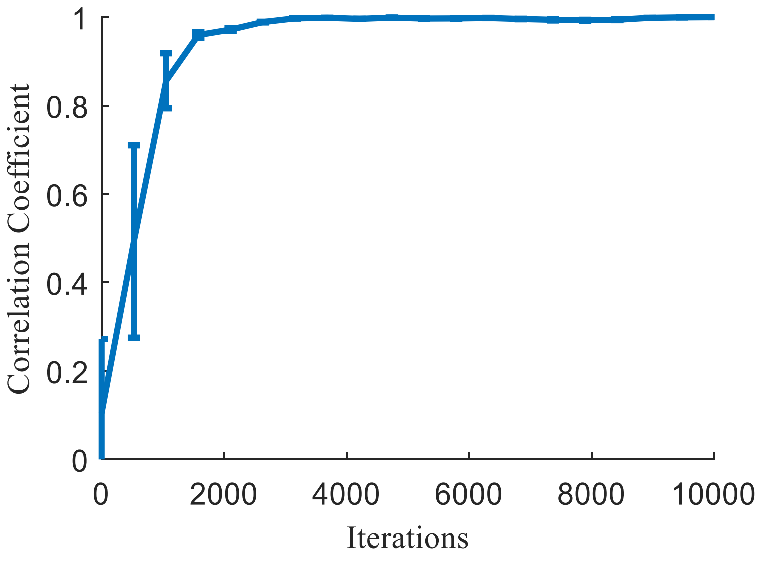

Figure: The correlation coefficient values between the LBAIM saliency maps generated by feature extractors learned at different stages and the saliency maps generated by feature extractors learned at the end of the training. We sampled the feature extractors at 20 equally spaced checkpoints during training. Error bars represent the standard deviation computed over 100 image samples.
